# Supplementary material for: Conserved molecular signatures in the spike protein provide evidence indicating the origin of SARS-CoV-2 and a Pangolin-CoV (MP789) by recombination(s) between specific lineages of Sarbecoviruses
Source: PeerJ. 2021 Nov 12;9:e12434. doi: 10.7717/peerj.12434 (PMC8592051; doi:10.7717/peerj.12434)
Supplement: Supplemental Information 2 [file peerj-09-12434-s002.pdf]

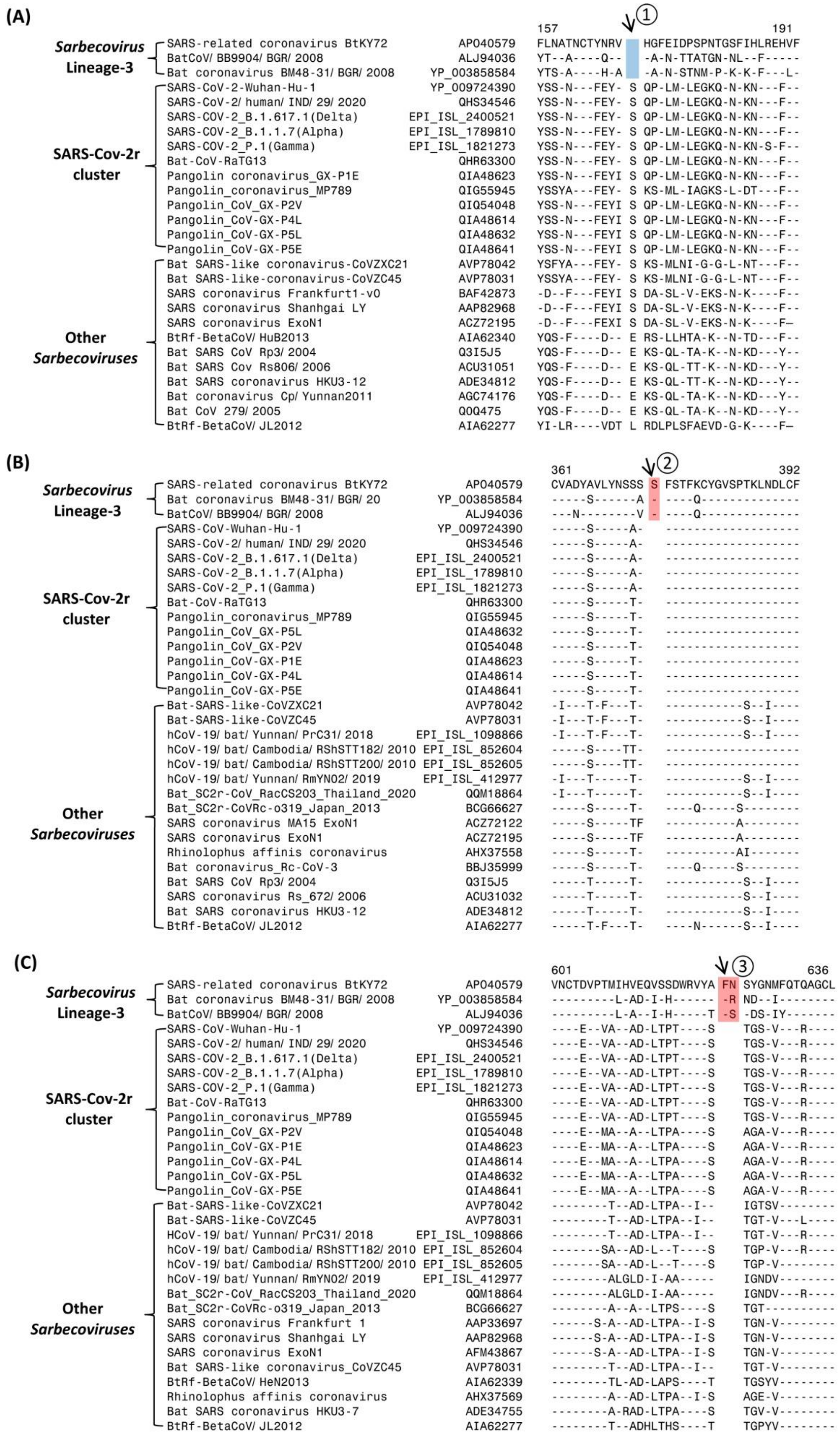

Figure S2. Excerpts from the sequence alignment for three regions of the spike protein depicting three CSIs which are specific for the CoVs from *Sarbecovirus* lineage-3
