# Supplementary material for: Conserved molecular signatures in the spike protein provide evidence indicating the origin of SARS-CoV-2 and a Pangolin-CoV (MP789) by recombination(s) between specific lineages of Sarbecoviruses
Source: PeerJ. 2021 Nov 12;9:e12434. doi: 10.7717/peerj.12434 (PMC8592051; doi:10.7717/peerj.12434)
Supplement: Supplemental Information 3 — (A) shows a 2 aa deletion (④) in the N-protein that is specific for the SARS-CoV-2r, CoVZC/PrC31 clusters as well as other Bat SARS-CoV-2 related strains of CoVs. The viruses from Sarbecovirus lineage-3 contain a separate 1 aa deletion (⑤) in this position. The (B) shows the sequence alignment of N-protein depicting a 2 aa deletion (⑥) that is specific for pangolin CoVs. A 1 aa deletion (⑦) specific for Sarbecovirus lineages-3 is also present in a neighboring region. [file peerj-09-12434-s003.pdf]

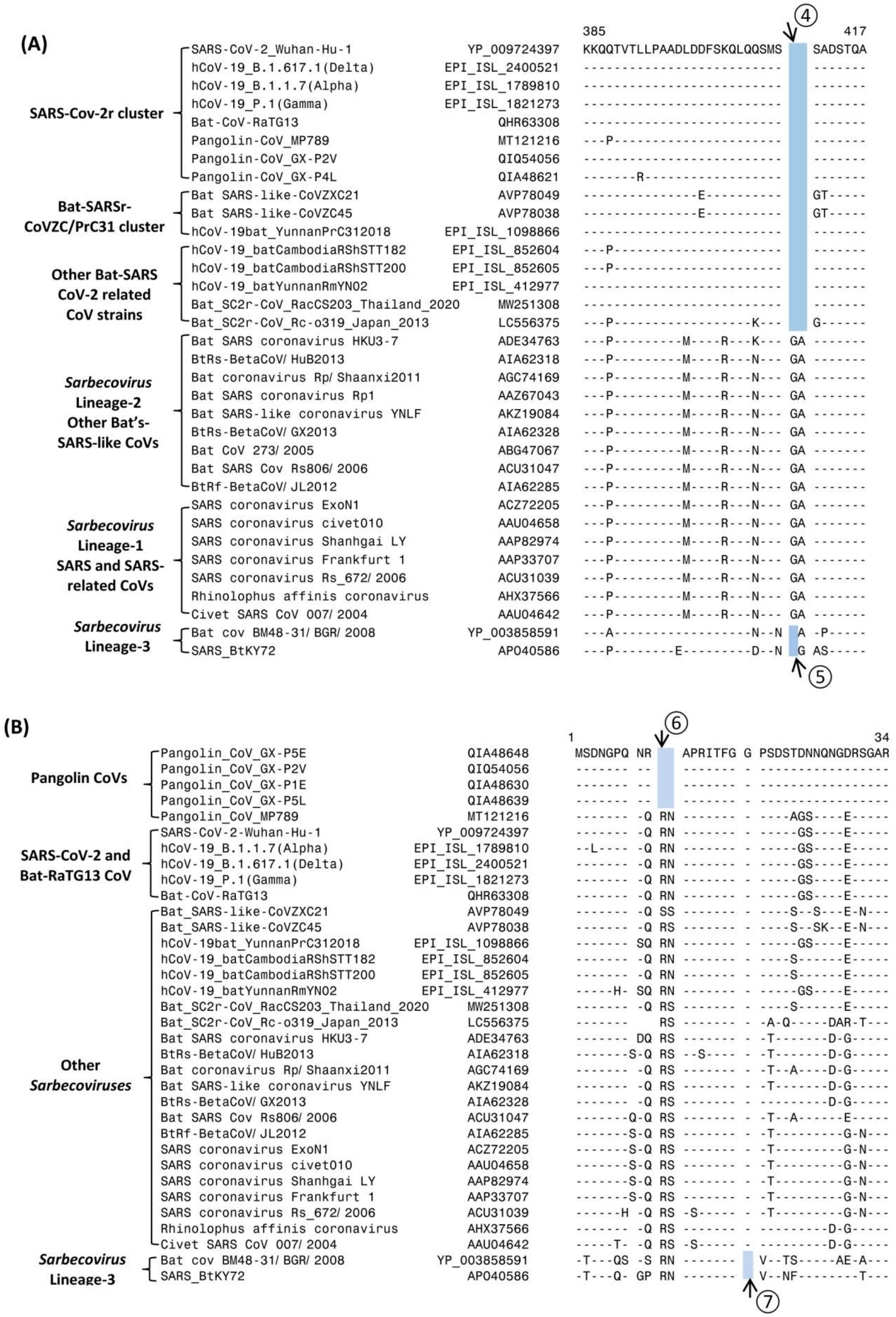

Figure S3. Panel (A) shows a 2 aa deletion (④) in the N-protein that is specific for the SARS-CoV-2r, CoVZC/PrC31 clusters as well as other Bat SARS-CoV-2 related strains of CoVs. The viruses from *Sarbecovirus* lineage-3 contain a separate 1 aa deletion (⑤) in this position. The panel (B) shows the sequence alignment of N-protein depicting a 2 aa deletion (⑥) that is specific for pangolin CoVs. A 1 aa deletion (⑦) specific for *Sarbecovirus* lineages-3 is also present in a neighboring region.
