# Supplementary material for: Conserved molecular signatures in the spike protein provide evidence indicating the origin of SARS-CoV-2 and a Pangolin-CoV (MP789) by recombination(s) between specific lineages of Sarbecoviruses
Source: PeerJ. 2021 Nov 12;9:e12434. doi: 10.7717/peerj.12434 (PMC8592051; doi:10.7717/peerj.12434)
Supplement: Supplemental Information 4 — In this alignment, the polymorphic positions where the sequence of pangolin CoV_MP789 is identical to that of CoVZC/PrC31 cluster of viruses are highlighted in yellow, whereas the polymorphic positions where the sequence of pangol is similar to SARS-CoV-2r viruses are highlighted in blue. Location containing Ssp1 restriction site is highlighted with red box and labelled. [file peerj-09-12434-s004.pdf]

Figure S4. A multiple sequence alignment of the spike protein from pangolin-CoV_MP789 and representative CoV strains from SARS-CoV-2 and CoVZC/Prc31 clusters showing the chimeric nature of MP789 sequence. In this alignment, the polymorphic positions where the sequence of pangolin CoV_MP789 is identical to that of CoVZC/PrC31 cluster of viruses are highlighted in yellow, whereas the polymorphic positions where the sequence of pangolin-CoV_MP789 is similar to SARS-CoV-2r viruses are highlighted in blue. Location containing Ssp1 restriction site is highlighted with red box and labelled.

S1-NTD

```
YP_009724390.1_SARS-COV-2/Wuhan-Hu-1                   M--FVFLVLLPLVSSQCVNLTTRTQLPPAYTNSFTRGVYYPDKVFRSSVLHSTQDLFLPF   58
Bat-CoV-RaTG13_QHR63300.2_MN996532.2                   M--FVFLVLLPLVSSQCVNLTTRTQLPPAYTNSSTRGVYYPDKVFRSSVLHLTQDLFLPF   58
Pangolin-CoV_MP789_QIG55945.1                          M-LFFFFLHFALVNSQCVNLTGRAAIQPSFTNSSQRGVYYPDTIFRSNTLVLSQGYFLPF   59
Bat-SARS-like-CoVZXC21_AVP78042.1                      M-LFFLFLQFALVNSQC-DLTGRTPLNPNYTNSSQRGVYYPDTIYRSDTLVLSQGYFLPF   58
Bat-SARS-like-CoVZC45_AVP78031.1_MG772933.1            M-LFFLFLQFALVNSQCVNLTGRTPLNPNYTNSSQRGVYYPDTIYRSDTLVLSQGYFLPF   59
hCoV-19/bat/Yunnan/PrC31/2018|EPI_ISL_1098866          M-LFFLFLQFTLVNSQCVNLTGRTPLNPNYTNSSQRGVYYPDTIYRSDTLVLSQGYFLPF   59
                                                       * :* ::: : *..***  ** *    *  ***  ******* ::**..*  :*. ****

YP_009724390.1_SARS-COV-2/Wuhan-Hu-1                   FSNVTWFHAIHVSGTNGTKRFDNPVLPFNDGVYFASTEKSNIIRGWIFGTTLDSKTQSLL   118
Bat-CoV-RaTG13_QHR63300.2_MN996532.2                   FSNVTWFHAIHVSGTNGIKRFDNPVLPFNDGVYFASTEKSNIIRGWIFGTTLDSKTQSLL   118
Pangolin-CoV_MP789_QIG55945.1                          YSNVSWYYALTKTN-SAEKRVDNPVLDFKDGIYFAATEKSNIVRGWIFGTTLDNTSQSLL   118
Bat-SARS-like-CoVZXC21_AVP78042.1                      YSNVSWYYSLTTNN-AATKRTDNPILDFKDGIYFAATEHSNIVRGWIFGTTLDNTSQSLL   117
Bat-SARS-like-CoVZC45_AVP78031.1_MG772933.1            YSNVSWYYSLTTNN-AATKRTDNPILDFKDGIYFAATEHSNIIRGWIFGTTLDNTSQSLL   118
hCoV-19/bat/Yunnan/PrC31/2018|EPI_ISL_1098866          YSNVSWYYSLTTNN-AATKRTDNPILDFKDGIYFAATEHSNIIRGWIFGTTLDNTSQSLL   118
                                                        ***:*:.:        *** ***:* * **:***:**:***:**********..:****

YP_009724390.1_SARS-COV-2/Wuhan-Hu-1                   IVNNATNVVIKVCEFQFCNDPFLGVYYHKNNKSWMESEFRVYSSANNCTFEYVSQPFLMD   178
Bat-CoV-RaTG13_QHR63300.2_MN996532.2                   IVNNATNVVIKVCEFQFCNDPFLGVYYHKNNKSWMESEFRVYSSANNCTFEYVSQPFLMD   178
Pangolin-CoV_MP789_QIG55945.1                          IVNNATNVIIKVCNFQFCYDPYLSGYYH-NNKTWSTREFAVYSSYANCTFEYVSKSFMLD   177
Bat-SARS-like-CoVZXC21_AVP78042.1                      IVNNATNVIIKVCNFDFCYDPYLSGYYH-NNKTWSIREFAVYSFYANCTFEYVSKSFMLN   176
Bat-SARS-like-CoVZC45_AVP78031.1_MG772933.1            IVNNATNVIIKVCNFDFCYDPYLSGYYH-NNKTWSIREFAVYSSYANCTFEYVSKSFMLN   177
hCoV-19/bat/Yunnan/PrC31/2018|EPI_ISL_1098866          IVNNATNVIIKVCNFDFCYDPYLSGYYH-NNKTWSIREFAVYSSYANCTFEYVSKSFMLN   177
                                                       ********:****:*:** :* *  *** ***:*   ** ***   ********: * ::

YP_009724390.1_SARS-COV-2/Wuhan-Hu-1                   LEGKQGNFKNLREFVFKNIDGYFKIYSKHTPINLVRDLPQGFSALEPLVDLPIGINITRF   238
Bat-CoV-RaTG13_QHR63300.2_MN996532.2                   LEGKQGNFKNLREFVFKNIDGYFKIYSKHTPINLVRDLPPGFSALEPLVDLPIGINITRF   238
Pangolin-CoV_MP789_QIG55945.1                          IAGKSGLFDTLREFVFRNVDGYFKIYSKYTPVNVNSNLPIGFSALEPLVEIPAGINITKF   237
Bat-SARS-like-CoVZXC21_AVP78042.1                      ISGNGGLFNTLREFVFRNVDGHFKIYSKFTPVNLNRGLPTGLSVLQPLVELPVSINITKF   236
Bat-SARS-like-CoVZC45_AVP78031.1_MG772933.1            ISGNGGLFNTLREFVFRNVDGHFKIYSKFTPVNLNRGLPTGLSVLQPLVELPVSINITKF   237
hCoV-19/bat/Yunnan/PrC31/2018|EPI_ISL_1098866          ISGNAGLFNTLREFVFRNVDGHFKIYSKFTPVNLNRGLPTGLSVLQPLVELPVSINITKF   237
                                                         *: * *. ******:* **.******.**:*: *.** *:*.*.***.:* .**** *

YP_009724390.1_SARS-COV-2/Wuhan-Hu-1                   QTLLALHRSYLTPGDSSSGWTAGAAAYYVGYLQPRTFLLKYNENGTITDAVDCALDPLSE   298
Bat-CoV-RaTG13_QHR63300.2_MN996532.2                   QTLLALHRSYLTPGDSSSGWTAGAAAYYVGYLQPRTFLLKYNENGTITDAVDCALDPLSE   298
Pangolin-CoV_MP789_QIG55945.1                          RTLLTIHRGDPMP---NNGWTVFSAAYYVGYLAPRTFMLNYNENGTITDAVDCALDPLSE   294
Bat-SARS-like-CoVZXC21_AVP78042.1                      RTLLTIHRGDPMS---NNGWTAFSAAYFVGYLKPRTFMLKYNENGTITDAVDCALDPLSE   293
Bat-SARS-like-CoVZC45_AVP78031.1_MG772933.1            RTLLTIHRGDPMP---NNGWTAFSAAYFVGYLKPRTFMLKYNENGTITDAVDCALDPLSE   294
hCoV-19/bat/Yunnan/PrC31/2018|EPI_ISL_1098866          RTLLTIHRGDPMP---NNGWTAFSAAYFVGYLKPRTFMLKYDENGTITDAVDCALDPLSE   294
                                                       :***::**    *   ..***  :***:**** ****:***:******************
```

Ssp1 site

S1-CTD

```
YP_009724390.1_SARS-COV-2/Wuhan-Hu-1                   TKCTLKSFTVEKGIYQTSNFRVQPTESIVRFPNITNLCPFGEVFNATRFASVYAWNRKRI   358
Bat-CoV-RaTG13_QHR63300.2_MN996532.2                   TKCTLKSFTVEKGIYQTSNFRVQPTDSIVRFPNITNLCPFGEVFNATTFASVYAWNRKRI   358
Pangolin-CoV_MP789_QIG55945.1                          AKCTLKSLTVEKGIYQTSNFRVQPTESIVRFPNITNLCPFGEVFNATTFASVYAWNRKRI   354
Bat-SARS-like-CoVZXC21_AVP78042.1                      TKCTLKSLSVQKGIYQTSNFRVQPTQSIVRFPNITNVCPFHKVFNATRFPSVYAWERTKI   353
Bat-SARS-like-CoVZC45_AVP78031.1_MG772933.1            TKCTLKSLTVQKGIYQTSNFRVQPTQSVVRFPNITNVCPFHKVFNATRFPSVYAWERTKI   354
hCoV-19/bat/Yunnan/PrC31/2018|EPI_ISL_1098866          TKCTLKSLTVQKGIYQTSNFRVQPTQSVVRFPNITNVCPFHKVFNATRFPSVYAWERTKI   354
                                                        ******:.* ************** *.********.*** .***** * *****.* .*

YP_009724390.1_SARS-COV-2/Wuhan-Hu-1                   SNCVADYSVLYNSASFSTFKCYGVSPTKLNDLCFTNVYADSFVIRGDEVRQIAPGQTGKI   418
Bat-CoV-RaTG13_QHR63300.2_MN996532.2                   SNCVADYSVLYNSTSFSTFKCYGVSPTKLNDLCFTNVYADSFVITGDEVRQIAPGQTGKI   418
Pangolin-CoV_MP789_QIG55945.1                          SNCVADYSVLYNSTSFSTFKCYGVSPTKLNDLCFTNVYADSFVVRGDEVRQIAPGQTGRI   414
Bat-SARS-like-CoVZXC21_AVP78042.1                      SDCIADYTVFYNSTSFSTFKCYGVSPSKLIDLCFTSVYADTFLIRFSEVRQVAPGQTGVI   413
Bat-SARS-like-CoVZC45_AVP78031.1_MG772933.1            SDCIADYTVFYNSTSFSTFKCYGVSPSKLIDLCFTSVYADTFLIRFSEVRQVAPGQTGVI   414
hCoV-19/bat/Yunnan/PrC31/2018|EPI_ISL_1098866          SDCIADYTVFYNSTSFSTFKCYGVSPSKLIDLCFTSVYADTFLIRFSEVRQVAPGQTGVI   414
                                                       *:*:***:*:***:************:** *****.****:*::  .****:****** *

YP_009724390.1_SARS-COV-2/Wuhan-Hu-1                   ADYNYKLPDDFTGCVIAWNSNNLDSKVGGNYNYLYRLFRKSNLKPFERDISTEIYQAGST   478
Bat-CoV-RaTG13_QHR63300.2_MN996532.2                   ADYNYKLPDDFTGCVIAWNSKHIDAKEGGNFNYLYRLFRKANLKPFERDISTEIYQAGSK   478
Pangolin-CoV_MP789_QIG55945.1                          ADYNYKLPDDFTGCVIAWNSNNLDSKVGGNYNYLYRLFRKSNLKPFERDISTEIYQAGST   474
Bat-SARS-like-CoVZXC21_AVP78042.1                      ADYNYKLPDDFTGCVIAWNTAKQDT-----GHYFYRSHRSTKLKPFERDLSSDEN-----   463
Bat-SARS-like-CoVZC45_AVP78031.1_MG772933.1            ADYNYKLPDDFTGCVIAWNTAKQDV-----GNYFYRSHRSTKLKPFERDLSSDEN-----   464
hCoV-19/bat/Yunnan/PrC31/2018|EPI_ISL_1098866          ADYNYKLPDDFTGCVIAWNTAKQDV-----GSYFYRSHRSTKLKPFERDLSSDEN-----   464
                                                       *******************:   *        * ** .*.: *******:*::
```

```
YP_009724390.1_SARS-COV-2/Wuhan-Hu-1                PCNGVEGFNCYFPLQSYGFQPTNGVGYQPYRVVVLSFELLHAPATVCGPKKSTNLVKNKC    538
Bat-CoV-RaTG13_QHR63300.2_MN996532.2                PCNGQTGLNCYYPLYRYGFYPTDGVGHQPYRVVVLSFELLNAPATVCGPKKSTNLVKNKC    538
Pangolin-CoV_MP789_QIG55945.1                       PCNGVEGFNCYFPLQSYGFHPTNGVGYQPYRVVVLSFELLKAPATVCGPKQSTNLVKNKC    534
Bat-SARS-like-CoVZXC21_AVP78042.1                   ---G------VRTLSTYDFNPNVPLEYQATRVVVLSFELLNAPATVCGPKLSTQLVKNQC    514
Bat-SARS-like-CoVZC45_AVP78031.1_MG772933.1         ---G------VRTLSTYDFNPNVPLEYQATRVVVLSFELLNAPATVCGPKLSTQLVKNQC    515
hCoV-19/bat/Yunnan/PrC31/2018|EPI_ISL_1098866       ---G------VRTLSTYDFNPNVPLEYQATRVVVLSFELLNAPATVCGPKLSTQLVKNQC    515
                                                       .         *  * *  .  : :*  **********:********* **:****:*

YP_009724390.1_SARS-COV-2/Wuhan-Hu-1                VNFNFNGLTGTGVLTESNKKFLPFQQFGRDIADTTDAVRDPQTLEILDITPCSFGGVSVI    598
Bat-CoV-RaTG13_QHR63300.2_MN996532.2                VNFNFNGLTGTGVLTESNKKFLPFQQFGRDIADTTDAVRDPQTLEILDITPCSFGGVSVI    598
Pangolin-CoV_MP789_QIG55945.1                       VNFNFNGLTGTGVLTESSKKFLPFQQFGRDIADTTDAVRDPQTLEILDITPCSFGGVSVI    594
Bat-SARS-like-CoVZXC21_AVP78042.1                   VNFNFNGLKGTGVLTDSSKRFQSFQQFGKDASDFIDSVRDPQTLEILDITPCSFGGVSVI    574
Bat-SARS-like-CoVZC45_AVP78031.1_MG772933.1         VNFNFNGLKGTGVLTDSSKRFQSFQQFGKDASDFIDSVRDPQTLEILDITPCSFGGVSVI    575
hCoV-19/bat/Yunnan/PrC31/2018|EPI_ISL_1098866       VNFNFNGLKGTGVLTDSSKKFQSFQQFGKDASDFIDSVRDPQTLEILDITPCSFGGVSVI    575
                                                    ********.******.*.*:*  *****:* :*  ::***********************

YP_009724390.1_SARS-COV-2/Wuhan-Hu-1                TPGTNTSNQVAVLYQDVNCTEVPVAIHADQLTPTWRVYSTGSNVFQTRAGCLIGAEHVNN    658
Bat-CoV-RaTG13_QHR63300.2_MN996532.2                TPGTNASNQVAVLYQDVNCTEVPVAIHADQLTPTWRVYSTGSNVFQTRAGCLIGAEHVNN    658
Pangolin-CoV_MP789_QIG55945.1                       TPGTNTSNQVAVLYQDVNCTEVPVAIHADQLTPTWRVYSTGSNVFQTRAGCLIGAEHVNN    654
Bat-SARS-like-CoVZXC21_AVP78042.1                   TPGTNTSSEVAVLYQDVNCTDVPTTIHADQLTPAWRIYAIGTSVFQTQAGCLIGAEHVNA    634
Bat-SARS-like-CoVZC45_AVP78031.1_MG772933.1         TPGTNTSLEVAVLYQDVNCTDVPTTIHADQLTPAWRIYATGTNVFQTQAGCLIGAEHVNA    635
hCoV-19/bat/Yunnan/PrC31/2018|EPI_ISL_1098866       TPGTNTSLEVAVLYQDVNCTDVPTTIHADQLTPAWRIYATGTNVFQTLAGCLIGAEHVNA    635
                                                    *****:* :***********:** :******: :**:*: * .**** ***********

YP_009724390.1_SARS-COV-2/Wuhan-Hu-1                SYECDIPIGAGICASYQTQTNSPRRARSVASQSIIAYTMSLGAENSVAYSNNSIAIPTNF    718
Bat-CoV-RaTG13_QHR63300.2_MN996532.2                SYECDIPIGAGICASYQTQTNS----RSVASQSIIAYTMSLGAENSVAYSNNSIAIPTNF    714
Pangolin-CoV_MP789_QIG55945.1                       TYECDIPIGAGICASYQTQTNS----RSVSSQAIIAYTMSLGAENSVAYANNSIAIPTNF    710
Bat-SARS-like-CoVZXC21_AVP78042.1                   SYECDIPIGAGICASYHTASIL----RSTGQKAIVAYTMSLGAENSIAYANNSIAIPTNF    690
Bat-SARS-like-CoVZC45_AVP78031.1_MG772933.1         SYECDIPIGAGICASYHTASIL----RSTSQKAIVAYTMSLGAENSIAYANNSIAIPTNF    691
hCoV-19/bat/Yunnan/PrC31/2018|EPI_ISL_1098866       SYECDIPIGAGICASYHTAPIL----RSTSQKAIVAYTMSLGAENSIAYANNSIAIPTNF    691
                                                    :***************:*        **. .::*:**********:**:**********

YP_009724390.1_SARS-COV-2/Wuhan-Hu-1                TISVTTEILPVSMTKTSVDCTMYICGDSTECSNLLLQYGSFCTQLNRALTGIAVEQDKNT    778
Bat-CoV-RaTG13_QHR63300.2_MN996532.2                TISVTTEILPVSMTKTSVDCTMYICGDSTECSNLLLQYGSFCTQLNRALTGIAVEQDKNT    774
Pangolin-CoV_MP789_QIG55945.1                       TISVTTEILPVSMTKTSVDCTMYICGDSIECSNLLLQYGSFCTQLNRALTGIAVEQDKNT    770
Bat-SARS-like-CoVZXC21_AVP78042.1                   SISVTTEVMPVSMAKTSVDCTMYICGDSIECSNLLLQYGSFCTQLNRALSGIAIEQDKNT    750
Bat-SARS-like-CoVZC45_AVP78031.1_MG772933.1         SISVTTEVMPVSMAKTSVDCTMYICGDSIECSNLLLQYGSFCTQLNRALSGIAIEQDKNT    751
hCoV-19/bat/Yunnan/PrC31/2018|EPI_ISL_1098866       SISVTTEVMPVSMAKTSVDCTMYICGDSIECSNLLLQYGSFCTQLNRALSGIAIEQDKNT    751
                                                    :******::****:***********************************:***:******

YP_009724390.1_SARS-COV-2/Wuhan-Hu-1                QEVFAQVKQIYKTPPIKDFGGFNFSQILPDPSKPSKRSFIEDLLFNKVTLADAGFIKQYG    838
Bat-CoV-RaTG13_QHR63300.2_MN996532.2                QEVFAQVKQIYKTPPIKDFGGFNFSQILPDPSKPSKRSFIEDLLFNKVTLADAGFIKQYG    834
Pangolin-CoV_MP789_QIG55945.1                       QEVFAQVKQIYKTPPIKDFGGFNFSQILPDPSKPSKRSFIEDLLFNKVTLADAGFIKQYG    830
Bat-SARS-like-CoVZXC21_AVP78042.1                   QEVFAQVKQIYKTPPIKDFGGFNFSQILPDPSKPSKRSFIEDLLFNKVTLADAGFIKQYG    810
Bat-SARS-like-CoVZC45_AVP78031.1_MG772933.1         QEVFAQVKQIYKTPPIKDFGGFNFSQILPDPSKPSKRSFIEDLLFNKVTLADAGFIKQYG    811
hCoV-19/bat/Yunnan/PrC31/2018|EPI_ISL_1098866       QEVFAQVKQIYKTPPIKDFGGFNFSQILPDPSKPSKRSFIEDLLFNKVTLADAGFIKQYG    811
                                                    ************************************************************

YP_009724390.1_SARS-COV-2/Wuhan-Hu-1                DCLGDIAARDLICAQKFNGLTVLPPLLTDEMIAQYTSALLAGTITSGWTFGAGAALQIPF    898
Bat-CoV-RaTG13_QHR63300.2_MN996532.2                DCLGDIAARDLICAQKFNGLTVLPPLLTDEMIAQYTSALLAGTITSGWTFGAGAALQIPF    894
Pangolin-CoV_MP789_QIG55945.1                       DCLGDIAARDLICAQKFNGLTVLPPLLTDEMIAQYTSALLAGTITSGWTFGAGAALQIPF    890
Bat-SARS-like-CoVZXC21_AVP78042.1                   DCLGDISARDLICAQKFNGLTVLPPLLTDEMIAAYTAALISGTATAGWTFGAGAALQIPF    870
Bat-SARS-like-CoVZC45_AVP78031.1_MG772933.1         DCLGGISARDLICAQKFNGLTVLPPLLTDEMIAAYTAALISGTATAGWTFGAGAALQIPF    871
hCoV-19/bat/Yunnan/PrC31/2018|EPI_ISL_1098866       DCLGGISARDLICAQKFNGLTVLPPLLTDEMIAAYTAALISGTATAGWTFGAGAALQIPF    871
                                                    ****.*:************************** **:**::** *:**************

YP_009724390.1_SARS-COV-2/Wuhan-Hu-1                AMQMAYRFNGIGVTQNVLYENQKLIANQFNSAIGKIQDSLSSTASALGKLQDVVNQNAQA    958
Bat-CoV-RaTG13_QHR63300.2_MN996532.2                AMQMAYRFNGIGVTQNVLYENQKLIANQFNSAIGKIQDSLSSTASALGKLQDVVNQNAQA    954
Pangolin-CoV_MP789_QIG55945.1                       AMQMAYRFNGIGVTQNVLYENQKLIANQFNSAIGKIQDSLSSTASALGKLQDVVNQNAQA    950
Bat-SARS-like-CoVZXC21_AVP78042.1                   AMQMAYRFNGIGVTQNVLYENQKLIANQFNSAIGKIQESLTSTASALGKLQDVVNQNAQA    930
Bat-SARS-like-CoVZC45_AVP78031.1_MG772933.1         AMQMAYRFNGIGVTQNVLYENQKLIANQFNSAIGKIQESLTSTASALGKLQDVVNQNAQA    931
hCoV-19/bat/Yunnan/PrC31/2018|EPI_ISL_1098866       AMQMAYRFNGIGVTQNVLYENQKLIANQFNSAIGKIQESLTSTASALGKLQDVVNQNAQA    931
                                                    *************************************:**:*******************

YP_009724390.1_SARS-COV-2/Wuhan-Hu-1                LNTLVKQLSSNFGAISSVLNDILSRLDKVEAEVQIDRLITGRLQSLQTYVTQQLIRAAEI    1018
Bat-CoV-RaTG13_QHR63300.2_MN996532.2                LNTLVKQLSSNFGAISSVLNDILSRLDKVEAEVQIDRLITGRLQSLQTYVTQQLIRAAEI    1014
Pangolin-CoV_MP789_QIG55945.1                       LNTLVKQLSSNFGAISSVLNDILSRLDKVEAEVQIDRLITGRLQSLQTYVTQQLIRAAEI    1010
Bat-SARS-like-CoVZXC21_AVP78042.1                   LNTLVKQLSSNFGAISSVLNDILSRLDKVEAEVQIDRLITGRLQSLQTYVTQQLIRAAEI    990
Bat-SARS-like-CoVZC45_AVP78031.1_MG772933.1         LNTLVKQLSSNFGAISSVLNDILSRLDKVEAEVQIDRLITGRLQSLQTYVTQQLIRAAEI    991
hCoV-19/bat/Yunnan/PrC31/2018|EPI_ISL_1098866       LNTLVKQLSSNFGAISSVLNDILSRLDKVEAEVQIDRLITGRLQSLQTYVTQQLIRAAEI    991
hCoV-19/bat/Cambodia/RShSTT182/2010|EPI_ISL_852604  LNTLVKQLSSNFGAISSVLNDILSRLDKVEAEVQIDRLITGRLQSLQTYVTQQLIRAAEI    996
hCoV-19/bat/Cambodia/RShSTT200/2010|EPI_ISL_852605  LNTLVKQLSSNFGAISSVLNDILSRLDKVEAEVQIDRLITGRLQSLQTYVTQQLIRAAEI    996
                                                    ************************************************************

YP_009724390.1_SARS-COV-2/Wuhan-Hu-1                RASANLAATKMSECVLGQSKRVDFCGKGYHLMSFPQSAPHGVVFLHVTYVPAQEKNFTTA    1078
Bat-CoV-RaTG13_QHR63300.2_MN996532.2                RASANLAATKMSECVLGQSKRVDFCGKGYHLMSFPQSAPHGVVFLHVTYVPAQEKNFTTA    1074
Pangolin-CoV_MP789_QIG55945.1                       RASANLAATKMSECVLGQSKRVDFCGKGYHLMSFPQSAPHGVVFLHVTYVPSQEKNFTTT    1070
Bat-SARS-like-CoVZXC21_AVP78042.1                   RASANLAATKMSECVLGQSKRVDFCGKGYHLMSFPQSAPHGVVFLHVTYIPSQEKNFTTA    1050
Bat-SARS-like-CoVZC45_AVP78031.1_MG772933.1         RASANLAATKMSECVLGQSKRVDFCGKGYHLMSFPQSAPHGVVFLHVTYIPSQEKNFTTA    1051
hCoV-19/bat/Yunnan/PrC31/2018|EPI_ISL_1098866       RASANLAATKMSECVLGQSKRVDFCGKGYHLMSFPQSAPHGVVFLHVTYIPSQEKNFTTA    1051
                                                    *************************************************:*:*:*****:
```

```
YP_009724390.1_SARS-COV-2/Wuhan-Hu-1                    PAICHDGKAHFPREGVFVSNGTHWFVTQRNFYEPQIITTDNTFVSGNCDVVIGIVNNTVY    1138
Bat-CoV-RaTG13_QHR63300.2_MN996532.2                    PAICHDGKAHFPREGVFVSNGTHWFVTQRNFYEPQIITTDNTFVSGSCDVVIGIVNNTVY    1134
Pangolin-CoV_MP789_QIG55945.1                           PAICHEGKAHFPREGVFVSNGTHWFVTQRNFYEPQIITTDNTFVSGSCDVVIGIVNNTVY    1130
Bat-SARS-like-CoVZXC21_AVP78042.1                       PAICHEGKAHFPREGVFVSNGTHWFVTQRNFYEPQIITTDNTFVSGNCDVVIGIINNTVY    1110
Bat-SARS-like-CoVZC45_AVP78031.1_MG772933.1             PAICHEGKAHFPREGVFVSNGTHWFVTQRNFYEPKIITTDNTFVSGNCDVVIGIINNTVY    1111
hCoV-19/bat/Yunnan/PrC31/2018|EPI_ISL_1098866           PAICHDGKAHFPREGVFVSNGTHWFITQRNFYEPQIITTDNTFVSGNCDVVIGIVNNTVY    1111
                                                        *****:*******************:********:*********** *******:*****


YP_009724390.1_SARS-COV-2/Wuhan-Hu-1                    DPLQPELDSFKEELDKYFKNHTSPDVDLGDISGINASVVNIQKEIDRLNEVAKNLNESLI    1198
Bat-CoV-RaTG13_QHR63300.2_MN996532.2                    DPLQPELDSFKEELDKYFKNHTSPDVDLGDISGINASVVNIQKEIDRLNEVAKNLNESLI    1194
Pangolin-CoV_MP789_QIG55945.1                           DPLQPELDSFKEELDKYFKNHTSPDVDLGDISGINASVVNIQKEIDRLNEVAKNLNESLI    1190
Bat-SARS-like-CoVZXC21_AVP78042.1                       DPLQPELDSFKEELDKYFKNHTSPDIDLGDISGINASVVNIQKEIDRLNEVARNLNESLI    1170
Bat-SARS-like-CoVZC45_AVP78031.1_MG772933.1             DPLQPELDSFKEELDKYFKNHTSPDIDLGDISGINASVVNIQKEIDRLNEVARNLNESLI    1171
hCoV-19/bat/Yunnan/PrC31/2018|EPI_ISL_1098866           DPLQPELDSFKEELDKYFKNHTSPDIDLGDISGINASVVNIQKEIDRLNEVARNLNESLI    1171
                                                        *************************:**************************:*******

YP_009724390.1_SARS-COV-2/Wuhan-Hu-1                    DLQELGKYEQYIKWPWYIWLGFIAGLIAIVMVTIMLCCMTSCCSCLKGCCSCGSCCKFDE    1258
Bat-CoV-RaTG13_QHR63300.2_MN996532.2                    DLQELGKYEQYIKWPWYIWLGFIAGLIAIIMVTIMLCCMTSCCSCLKGCCSCGSCCKFDE    1254
Pangolin-CoV_MP789_QIG55945.1                           DLQELGKYEQYIKWPWYIWLGFIAGLIAIIMVTIMLCCMTSCCSCLKGCCSCGSCCKFDE    1250
Bat-SARS-like-CoVZXC21_AVP78042.1                       DLQELGKYEHYIKWPWYVWLGFIAGLIAIVMVTILLCCMTSCCSCLKGCCSCGFCCKFDE    1230
Bat-SARS-like-CoVZC45_AVP78031.1_MG772933.1             DLQELGKYEQYIKWPWYVWLGFIAGLIAIVMVTILLCCMTSCCSCLKGCCSCGSCCKFDE    1231
hCoV-19/bat/Yunnan/PrC31/2018|EPI_ISL_1098866           DLQELGKYEQYIKWPWYVWLGFIAGLIAIVMVTILLCCMTSCCSCLKGCCSCGSCCKFDE    1231
                                                        *********:*******:***********:****:****************** ******

YP_009724390.1_SARS-COV-2/Wuhan-Hu-1                    DDSEPVLKGVKLHYT     1273
Bat-CoV-RaTG13_QHR63300.2_MN996532.2                    DDSEPVLKGVKLHYT     1269
Pangolin-CoV_MP789_QIG55945.1                           DDSEPVLKGVKLHYT     1265
Bat-SARS-like-CoVZXC21_AVP78042.1                       DDSEPVLKGVKLHYT     1245
Bat-SARS-like-CoVZC45_AVP78031.1_MG772933.1             DDSEPVLKGVKLHYT     1246
hCoV-19/bat/Yunnan/PrC31/2018|EPI_ISL_1098866           DDSEPVLKGVKLHYT     1246
                                                        ***************
```
