# Supplementary material for: Conserved molecular signatures in the spike protein provide evidence indicating the origin of SARS-CoV-2 and a Pangolin-CoV (MP789) by recombination(s) between specific lineages of Sarbecoviruses
Source: PeerJ. 2021 Nov 12;9:e12434. doi: 10.7717/peerj.12434 (PMC8592051; doi:10.7717/peerj.12434)
Supplement: Supplemental Information 5 — In this alignment, the polymorphic positions where the sequences of SARS-CoV-2r viruses are identical to that of CoVZC/PrC31 cluster of viruses are highlighted in yellow, whereas the polymorphic positions where the sequence of RShSTT182/200 and SARS coronavirus ExoN1 viruses are similar to SARS-CoV-2r viruses are highlighted in blue. Location containing Ssp1 restriction site is highlighted with red box and labelled. [file peerj-09-12434-s005.pdf]

Figure S5. A multiple sequence alignment of the spike protein from SARS coronavirus ExoN1, SARS coronavirus ShanghaiQXC2 and representative CoV strains from SARS-CoV-2r, RShSTT182/200 and CoVZC/PrC31 clusters showing the chimeric nature of SARS-CoV-2 sequence. In this alignment, the polymorphic positions where the sequences of SARS-CoV-2r viruses are identical to that of CoVZC/PrC31 cluster of viruses are highlighted in yellow, whereas the polymorphic positions where the sequence of RShSTT182/200 and SARS coronavirus ExoN1 viruses are similar to SARS-CoV-2r viruses are highlighted in blue. Location containing Ssp1 restriction site is highlighted with red box and labelled.

S1-NTD

```
YP_009724390.1_SARS-COV-2/ Wuhan-Hu-1                    MFVFLVLLPLVS----SQCVNLTTRTQLPPAYTNSFTRGVYYPDKVFRSSVLHSTQDLFL    56
Bat-CoV-RaTG13_QHR63300.2_MN996532.2                     MFVFLVLLPLVS----SQCVNLTTRTQLPPAYTNSSTRGVYYPDKVFRSSVLHLTQDLFL    56
Bat-SARS-like-CoVZC45_AVP78031.1_MG772933.1              M-LFFLFLQFAL--VNSQCVNLTGRTPLNPNYTNSSQRGVYYPDTIYRSDTLVLSQGYFL    57
hCoV-19/ bat/ Yunnan/ PrC31/ 2018| EPI_ISL_1098866       M-LFFLFLQFTL--VNSQCVNLTGRTPLNPNYTNSSQRGVYYPDTIYRSDTLVLSQGYFL    57
hCoV-19/ bat/ Cambodia/ RShSTT182/ 2010| EPI_ISL_852604  MILLAFLIPLVT--AQQGCGVISTKPQPKAMTFLSSSRGVYYNDDIFRSSVLHLTQDYFL    58
hCoV-19/ bat/ Cambodia/ RShSTT200/ 2010| EPI_ISL_852605  MILLAFLIPLVT--AQQGCGVISTKPQPKAMTFLSSSRGVYYNDDIFRSSVLHLTQDYFL    58
AGT21078.1_SARS_coronavirus_ExoN1                        MFIFLLFLTLTSGSDLDRCTTFDDVQAPNYTQHTSSMRGVYYPDEIFRSDTLYLTQDLFL    60
AAR86775.1_SARS_coronavirus_ShanghaiQXC2                 MFIFLLFLTLTSGSDLDRCTTFDDVQAPNYTQHTSSMRGVYYPDEIFRSDTLYLTQDLFL    60
                                                         *::: .:: :.     . *  :            *  ***** * ::**..*  :*. **

YP_009724390.1_SARS-COV-2/ Wuhan-Hu-1                    PFFSNVTWFHAIHVSGTNGTKRFDNPVLPFNDGVYFASTEKSNIIRGWIFGTTLDSKTQS    116
Bat-CoV-RaTG13_QHR63300.2_MN996532.2                     PFFSNVTWFHAIHVSGTNGIKRFDNPVLPFNDGVYFASTEKSNIIRGWIFGTTLDSKTQS    116
Bat-SARS-like-CoVZC45_AVP78031.1_MG772933.1              PFYSNVSWYYSLTTN-NAATKRTDNPILDFKDGIYFAATEHSNIIRGWIFGTTLDNTSQS    116
hCoV-19/ bat/ Yunnan/ PrC31/ 2018| EPI_ISL_1098866       PFYSNVSWYYSLTTN-NAATKRTDNPILDFKDGIYFAATEHSNIIRGWIFGTTLDNTSQS    116
hCoV-19/ bat/ Cambodia/ RShSTT182/ 2010| EPI_ISL_852604  PFNSTLTQYFSHV---IDTSSYFDNPILNFGDGVYFASTEKSNVIRGWIFGSSFDNTTQS    115
hCoV-19/ bat/ Cambodia/ RShSTT200/ 2010| EPI_ISL_852605  PFNSTLTQYFSHV---IDTSSYFDNPILNFGDGVYFASTEKSNVIRGWIFGSSFDNTTQS    115
AGT21078.1_SARS_coronavirus_ExoN1                        PFYSNVTGFHTIN-------HTFGNPVIPFKDGIYFAATEKSNVVRGWVFGSTMNNKSQS    113
AAR86775.1_SARS_coronavirus_ShanghaiQXC2                 PFYSNVTGFHTIN-------HTFGNPVIPFKDGIYFAATEKSNVVRGWVFGSTMNNKSQS    113
                                                         ** *.:: :.:            .**:: * **:***:**:**::***:**::::..:**

YP_009724390.1_SARS-COV-2/ Wuhan-Hu-1                    LLIVNNATNVVIKVCEFQFCNDPFLGVYYHKNNKSWMESEFRVYSSANNCTFEYVSQPFL    176
Bat-CoV-RaTG13_QHR63300.2_MN996532.2                     LLIVNNATNVVIKVCEFQFCNDPFLGVYYHKNNKSWMESEFRVYSSANNCTFEYVSQPFL    176
Bat-SARS-like-CoVZC45_AVP78031.1_MG772933.1              LLIVNNATNVIIKVCNFDFCYDPYLSGYYHN-NKTWSIREFAVYSSYANCTFEYVSKSFM    175
hCoV-19/ bat/ Yunnan/ PrC31/ 2018| EPI_ISL_1098866       LLIVNNATNVIIKVCNFDFCYDPYLSGYYHN-NKTWSIREFAVYSSYANCTFEYVSKSFM    175
hCoV-19/ bat/ Cambodia/ RShSTT182/ 2010| EPI_ISL_852604  AIIVNNSTHVIIRVCNFNLCKEPMYTVSGGVQKDSW------VYQSAFNCTYDRVEKSFQ    169
hCoV-19/ bat/ Cambodia/ RShSTT200/ 2010| EPI_ISL_852605  AIIVNNSTHVIIRVCNFNLCKEPMYTVSGGVQKDSW------VYQSAFNCTYDRVEKSFQ    169
AGT21078.1_SARS_coronavirus_ExoN1                        VIIINNSTNVVIRACNFELCDNPFFAVSKPMGTQ----THTMIFDNAFNCTFEYISDAFS    169
AAR86775.1_SARS_coronavirus_ShanghaiQXC2                 VIIINNSTNVVIRACNFELCDNPFFAVSKPMGTQ----THTMIFDNAFNCTFEYISDAFS    169
                                                          :*:**:*:*:*:.*:*::* :*         ..        ::.   ***:: :.. *

YP_009724390.1_SARS-COV-2/ Wuhan-Hu-1                    MDLEGKQGNFKNLREFVFKNIDGYFKIYSKHTPINLVRDLPQGFSALEPLVDLPIGINIT    236
Bat-CoV-RaTG13_QHR63300.2_MN996532.2                     MDLEGKQGNFKNLREFVFKNIDGYFKIYSKHTPINLVRDLPPGFSALEPLVDLPIGINIT    236
Bat-SARS-like-CoVZC45_AVP78031.1_MG772933.1              LNISGNGGLFNTLREFVFRNVDGHFKIYSKFTPVNLNRGLPTGLSVLQPLVELPVSINIT    235
hCoV-19/ bat/ Yunnan/ PrC31/ 2018| EPI_ISL_1098866       LNISGNAGLFNTLREFVFRNVDGHFKIYSKFTPVNLNRGLPTGLSVLQPLVELPVSINIT    235
hCoV-19/ bat/ Cambodia/ RShSTT182/ 2010| EPI_ISL_852604  LDTTVKTGNFKDLREYVFKNRDGFLSVYYSYTPVDLLRGLPSGFSVLRPILKLPLGLNIT    229
hCoV-19/ bat/ Cambodia/ RShSTT200/ 2010| EPI_ISL_852605  LDTTVKTGNFKDLREYVFKNRDGFLSVYYSYTPVDLLRGLPSGFSVLRPILKLPLGLNIT    229
AGT21078.1_SARS_coronavirus_ExoN1                        LDVSEKSGNFKHLREFVFKNKDGFLYVYKGYQPIDVVRDLPSGFNTLKPIFKLPLGINIT    229
AAR86775.1_SARS_coronavirus_ShanghaiQXC2                 LDVSEKSGNFKHLREFVFKNKDGFLYVYKGYQPIDVVRDLPSGFNTLKPIFKLPLGINIT    229
                                                         ::   : * *: ***:**:* **.: :*  . *::: *.** *:..*.*:..**:.:***

YP_009724390.1_SARS-COV-2/ Wuhan-Hu-1                    RFQTLLALHRSYLTPGDSSSGWTAGAAAYYVGYLQPRTFLLKYNENGTITDAVDCALDPL    296
Bat-CoV-RaTG13_QHR63300.2_MN996532.2                     RFQTLLALHRSYLTPGDSSSGWTAGAAAYYVGYLQPRTFLLKYNENGTITDAVDCALDPL    296
Bat-SARS-like-CoVZC45_AVP78031.1_MG772933.1              KFRTLLTIHRGDPM---PNNGWTAFSAAYFVGYLKPRTFMLKYNENGTITDAVDCALDPL    292
hCoV-19/ bat/ Yunnan/ PrC31/ 2018| EPI_ISL_1098866       KFRTLLTIHRGDPM---PNNGWTAFSAAYFVGYLKPRTFMLKYDENGTITDAVDCALDPL    292
hCoV-19/ bat/ Cambodia/ RShSTT182/ 2010| EPI_ISL_852604  SYRVVMAMFS------KTSSNFIPESAAYYTGNLKFSTFMLSFDENGTITQAVDCSQHPL    283
hCoV-19/ bat/ Cambodia/ RShSTT200/ 2010| EPI_ISL_852605  SYRVVMAMFS------KTSSNFIPESAAYYTGNLKFSTFMLSFDENGTITQAVDCSQHPL    283
AGT21078.1_SARS_coronavirus_ExoN1                        NFRAILTAFSP------AQDIWGTSAAAYFVGYLKPTTFMLKYDENGTITDAVDCSQNPL    283
AAR86775.1_SARS_coronavirus_ShanghaiQXC2                 NFRAILTAFSP------AQDIWGTSAAAYFVGYLKPTTFMLKYDENGTITDAVDCSQNPL    283
                                                         [illegible]

YP_009724390.1_SARS-COV-2/ Wuhan-Hu-1                    SETKCTLKSFTVEKGIYQTSNFRVQPTESIVRFPNITNLCPFGEVFNATRFASVYAWNRK    356
Bat-CoV-RaTG13_QHR63300.2_MN996532.2                     SETKCTLKSFTVEKGIYQTSNFRVQPTDSIVRFPNITNLCPFGEVFNATTFASVYAWNRK    356
Bat-SARS-like-CoVZC45_AVP78031.1_MG772933.1              SETKCTLKSLTVQKGIYQTSNFRVQPTQSVVRFPNITNVCPFHKVFNATRFPSVYAWERT    352
hCoV-19/ bat/ Yunnan/ PrC31/ 2018| EPI_ISL_1098866       SETKCTLKSLTVQKGIYQTSNFRVQPTQSVVRFPNITNVCPFHKVFNATRFPSVYAWERT    352
hCoV-19/ bat/ Cambodia/ RShSTT182/ 2010| EPI_ISL_852604  AELKCTLKSLNVVKGIYQTSNFRTSPTTQVVRFPNITNLCPFGEVFNATTFASVYAWNRR    343
hCoV-19/ bat/ Cambodia/ RShSTT200/ 2010| EPI_ISL_852605  AELKCTLKSLNVVKGIYQTSNFRTSPTTQVVRFPNITNLCPFGEVFNATTFASVYAWNRR    343
AGT21078.1_SARS_coronavirus_ExoN1                        AELKCSVKSFEIDKGIYQTSNFRVVPSGDVVRFPNITNLCPFGEVFNATKFPSVYAWERK    343
AAR86775.1_SARS_coronavirus_ShanghaiQXC2                 AELKCSVKSFEIDKGIYQTSNFRVVPSGDVVRFPNITNLCPFGEVFNATKFPSVYAWERK    343
                                                         :* **::**: : **********. *: .:********:*** :***** * *****:*
```

Ssp1 site

S1-CTD

```
YP_009724390.1_SARS-COV-2/ Wuhan-Hu-1                  RISNCVADYSVLYNSASFSTFKCYGVSPTKLNDLCFTNVYADSFVIRGDEVRQIAPGQTG   416
Bat-CoV-RaTG13_QHR63300.2_MN996532.2                   RISNCVADYSVLYNSTSFSTFKCYGVSPTKLNDLCFTNVYADSFVITGDEVRQIAPGQTG   416
Bat-SARS-like-CoVZC45_AVP78031.1_MG772933.1            KISDCIADYTVFYNSTSFSTFKCYGVSPSKLIDLCFTSVYADTFLIRFSEVRQVAPGQTG   412
hCoV-19/ bat/ Yunnan/ PrC31/ 2018| EPI_ISL_1098866     KISDCIADYTVFYNSTSFSTFKCYGVSPSKLIDLCFTSVYADTFLIRFSEVRQVAPGQTG   412
hCoV-19/ bat/ Cambodia/ RShSTT182/ 2010| EPI_ISL_852604 RISNCVADYSVLYNTTSFSTFKCYGVSPTKLNDLCFTNVYADSFVVRGDEVRQIAPGQTG   403
hCoV-19/ bat/ Cambodia/ RShSTT200/ 2010| EPI_ISL_852605 RISNCVADYSVLYNTTSFSTFKCYGVSPTKLNDLCFTNVYADSFVVRGDEVRQIAPGQTG   403
AGT21078.1_SARS_coronavirus_ExoN1                      KISNCVADYSVLYNSTFFSTFKCYGVSATKLNDLCFSNVYADSFVVKGDDVRQIAPGQTG   403
AAR86775.1_SARS_coronavirus_ShanghaiQXC2               KISNCVADYSVLYNSTFFSTFKCYGVSATKLNDLCFSNVYADSFVVKGDDVRQIAPGQTG   403
                                                       :**:*:***:*:**:: ********** :** ****:.****:*::  .:***:******

YP_009724390.1_SARS-COV-2/ Wuhan-Hu-1                  KIADYNYKLPDDFTGCVIAWNSNNLDSKVGGNYNYLYRLFRKSNLKPFERDISTEIYQAG   476
Bat-CoV-RaTG13_QHR63300.2_MN996532.2                   KIADYNYKLPDDFTGCVIAWNSKHIDAKEGGNFNYLYRLFRKANLKPFERDISTEIYQAG   476
Bat-SARS-like-CoVZC45_AVP78031.1_MG772933.1            VIADYNYKLPDDFTGCVIAWNTAKQD-----VGNYFYRSHRSTKLKPFERDLSSDEN---   464
hCoV-19/ bat/ Yunnan/ PrC31/ 2018| EPI_ISL_1098866     VIADYNYKLPDDFTGCVIAWNTAKQD-----VGSYFYRSHRSTKLKPFERDLSSDEN---   464
hCoV-19/ bat/ Cambodia/ RShSTT182/ 2010| EPI_ISL_852604 KIADYNYKLPDDFMGCVIAWNSISLDA--G--GSYYYRLFRKSVLKPFERDISTQLYQAG   459
hCoV-19/ bat/ Cambodia/ RShSTT200/ 2010| EPI_ISL_852605 KIADYNYKLPDDFMGCVIAWNSISLDA--G--GSYYYRLFRKSVLKPFERDISTQLYQAG   459
AGT21078.1_SARS_coronavirus_ExoN1                      VIADYNYKLPDDFMGCVLAWNTRNIDATSTGNYNYKYRSLRHGKLRPFERDISNVPFSPD   463
AAR86775.1_SARS_coronavirus_ShanghaiQXC2               VIADYNYKLPDDFMGCVLAWNTRNIDATSTGNYNYKYRYLRHGKLRPFERDISNVPFSPD   463
                                                        ************ ***:***:   *        * **  *   *:*****:*.

YP_009724390.1_SARS-COV-2/ Wuhan-Hu-1                  STPCNGVEGFNCYFPLQSYGFQPTNGVGYQPYRVVVLSFELLHAPATVCGPKKSTNLVKN   536
Bat-CoV-RaTG13_QHR63300.2_MN996532.2                   SKPCNGQTGLNCYYPLYRYGFYPTDGVGHQPYRVVVLSFELLNAPATVCGPKKSTNLVKN   536
Bat-SARS-like-CoVZC45_AVP78031.1_MG772933.1            -----------GVRTLSTYDFNPNVPLEYQATRVVVLSFELLNAPATVCGPKLSTQLVKN   513
hCoV-19/ bat/ Yunnan/ PrC31/ 2018| EPI_ISL_1098866     -----------GVRTLSTYDFNPNVPLEYQATRVVVLSFELLNAPATVCGPKLSTQLVKN   513
hCoV-19/ bat/ Cambodia/ RShSTT182/ 2010| EPI_ISL_852604 DKPCS-VEGPDCYYPLQSYYFQSTNGVGYQPYRVVVLSFELLNAPATVCGPKKSTHLVVN   518
hCoV-19/ bat/ Cambodia/ RShSTT200/ 2010| EPI_ISL_852605 DKPCS-VEGPDCYYPLQSYYFQSTNGVGYQPYRVVVLSFELLNAPATVCGPKKSTHLVVN   518
AGT21078.1_SARS_coronavirus_ExoN1                      GKPCTP-PALNCYWPLNDYGFYTTTGIGYQPYRVVVLSFELLNAPATVCGPKLSTDLIKN   522
AAR86775.1_SARS_coronavirus_ShanghaiQXC2               GKPCTP-PALNCYWPLNDYGFYTTTGIGYQPYRVVVLSFELLNAPATVCGPKLSTDLIKN   522
                                                                      *  * *  .  : :*  **********:********* **.*: *

YP_009724390.1_SARS-COV-2/ Wuhan-Hu-1                  KCVNFNFNGLTGTGVLTESNKKFLPFQQFGRDIADTTDAVRDPQTLEILDITPCSFGGVS   596
Bat-CoV-RaTG13_QHR63300.2_MN996532.2                   KCVNFNFNGLTGTGVLTESNKKFLPFQQFGRDIADTTDAVRDPQTLEILDITPCSFGGVS   596
Bat-SARS-like-CoVZXC21_AVP78042.1                      QCVNFNFNGLKGTGVLTDSSKRFQSFQQFGKDASDFIDSVRDPQTLEILDITPCSFGGVS   572
Bat-SARS-like-CoVZC45_AVP78031.1_MG772933.1            QCVNFNFNGLKGTGVLTDSSKRFQSFQQFGKDASDFIDSVRDPQTLEILDITPCSFGGVS   573
hCoV-19/ bat/ Yunnan/ PrC31/ 2018| EPI_ISL_1098866     QCVNFNFNGLKGTGVLTDSSKKFQSFQQFGKDASDFIDSVRDPQTLEILDITPCSFGGVS   573
hCoV-19/ bat/ Cambodia/ RShSTT182/ 2010| EPI_ISL_852604 KCVNFNFNGLTGTGVLTSSTKKFLPFQQFGRDVADTTNAVRDPQTLEVLDITPCSFGGVS   578
hCoV-19/ bat/ Cambodia/ RShSTT200/ 2010| EPI_ISL_852605 KCVNFNFNGLTGTGVLTSSTKKFLPFQQFGRDVADTTNAVRDPQTLEVLDITPCSFGGVS   578
AGT21078.1_SARS_coronavirus_ExoN1                      QCVNFNFNGLTGTGVLTPSSKRFQPFQQFGRDVSDFTDSVRDPKTSEILDISPCSFGGVS   582
AAR86775.1_SARS_coronavirus_ShanghaiQXC2               QCVNFNFNGLTGTGVLTPSSKRFQPFQQFGRDVSDFTDSVRDPKTSEILDISPCAFGGVS   582
                                                       :*********.****** *.*:*  *****:* :*  ::****:* *:***:**:*****

YP_009724390.1_SARS-COV-2/ Wuhan-Hu-1                  VITPGTNTSNQVAVLYQDVNCTEVPVAIHADQLTPTWRVYSTGSNVFQTRAGCLIGAEHV   656
Bat-CoV-RaTG13_QHR63300.2_MN996532.2                   VITPGTNASNQVAVLYQDVNCTEVPVAIHADQLTPTWRVYSTGSNVFQTRAGCLIGAEHV   656
Bat-SARS-like-CoVZC45_AVP78031.1_MG772933.1            VITPGTNTSLEVAVLYQDVNCTDVPTTIHADQLTPAWRIYATGTNVFQTQAGCLIGAEHV   633
hCoV-19/ bat/ Yunnan/ PrC31/ 2018| EPI_ISL_1098866     VITPGTNTSLEVAVLYQDVNCTDVPTTIHADQLTPAWRIYATGTNVFQTLAGCLIGAEHV   633
hCoV-19/ bat/ Cambodia/ RShSTT182/ 2010| EPI_ISL_852604 VITPGTNTSTQVAVLYQDVNCTDVPSAIHADQLSSTWRVYSTGPNVFQTRAGCLIGAEHV   638
hCoV-19/ bat/ Cambodia/ RShSTT200/ 2010| EPI_ISL_852605 VITPGTNTSTQVAVLYQDVNCTDVPSAIHADQLSSTWRVYSTGPNVFQTRAGCLIGAEHV   638
AGT21078.1_SARS_coronavirus_ExoN1                      VITPGTNASSEVAVLYQDVNCTDVSTAIHADQLTPAWRIYSTGNNVFQTQAGCLIGAEYV   642
AAR86775.1_SARS_coronavirus_ShanghaiQXC2               VITPGTNASSEVAVLYQDVNCTNVSAAIHADQLTPAWRIYSTGNNVFQTQAGCLIGAEHV   642
                                                       *******:* :***********:*  :******: :**:*: * .**** ********:*

YP_009724390.1_SARS-COV-2/ Wuhan-Hu-1                  NNSYECDIPIGAGICASYQTQTNSPRRARSVASQSIIAYTMSLGAENSVAYSNNSIAIPT   716
Bat-CoV-RaTG13_QHR63300.2_MN996532.2                   NNSYECDIPIGAGICASYQTQTNS----RSVASQSIIAYTMSLGAENSVAYSNNSIAIPT   712
Bat-SARS-like-CoVZC45_AVP78031.1_MG772933.1            NASYECDIPIGAGICASYHTASIL----RSTSQKAIVAYTMSLGAENSIAYANNSIAIPT   689
hCoV-19/ bat/ Yunnan/ PrC31/ 2018| EPI_ISL_1098866     NASYECDIPIGAGICASYHTAPIL----RSTSQKAIVAYTMSLGAENSIAYANNSIAIPT   689
hCoV-19/ bat/ Cambodia/ RShSTT182/ 2010| EPI_ISL_852604 NNSYDCDIPIGAGICASYQTQTNS----RSVTSQSIIAYTMSLGAENSVAYSNNSIAIPT   694
hCoV-19/ bat/ Cambodia/ RShSTT200/ 2010| EPI_ISL_852605 NNSYDCDIPIGAGICASYQTQTNS----RSVTSQSIIAYTMSLGAENSVAYSNNSIAIPT   694
AGT21078.1_SARS_coronavirus_ExoN1                      DTSYECDIPIGAGICASYHTVSLL----RSTSQKSIVAYTMSLGADSSIAYSNNTIAIPT   698
AAR86775.1_SARS_coronavirus_ShanghaiQXC2               DTSYECDIPIGAGICASYHTVSLL----RSTSQKSIVAYTMSLGADSSIAYSNNTIAIPT   698
                                                       : **:*************:*        **. .::*:********:.*:**:**:*****

YP_009724390.1_SARS-COV-2/ Wuhan-Hu-1                  NFTISVTTEILPVSMTKTSVDCTMYICGDSTECSNLLLQYGSFCTQLNRALTGIAVEQDK   776
Bat-CoV-RaTG13_QHR63300.2_MN996532.2                   NFTISVTTEILPVSMTKTSVDCTMYICGDSTECSNLLLQYGSFCTQLNRALTGIAVEQDK   772
Bat-SARS-like-CoVZC45_AVP78031.1_MG772933.1            NFSISVTTEVMPVSMAKTSVDCTMYICGDSIECSNLLLQYGSFCTQLNRALSGIAIEQDK   749
hCoV-19/ bat/ Yunnan/ PrC31/ 2018| EPI_ISL_1098866     NFSISVTTEVMPVSMAKTSVDCTMYICGDSIECSNLLLQYGSFCTQLNRALSGIAIEQDK   749
hCoV-19/ bat/ Cambodia/ RShSTT182/ 2010| EPI_ISL_852604 NFTISVTTEILPVSMTKTSVDCTMYICGDSTECSNLLLQYGSFCSQLNRALTGIAVEQDK   754
hCoV-19/ bat/ Cambodia/ RShSTT200/ 2010| EPI_ISL_852605 NFTISVTTEILPVSMTKTSVDCTMYICGDSTECSNLLLQYGSFCSQLNRALTGIAVEQDK   754
AGT21078.1_SARS_coronavirus_ExoN1                      NFSISIATEVMPVSMAKTSVDCNMYICGDSTECANLLLQYGSFCTQLNRALSGIAAEQDR   758
AAR86775.1_SARS_coronavirus_ShanghaiQXC2               NFSISITTEVMPVSMAKTSVDCNMYICGDSTECANLLLQYGSFCTQLNRALSGIAAEQDR   758
                                                       **:**::**::****:******.******* **:**********:******:*** ***:

YP_009724390.1_SARS-COV-2/ Wuhan-Hu-1                  NTQEVFAQVKQIYKTPPIKDFGGFNFSQILPDPSKPSKRSFIEDLLFNKVTLADAGFIKQ   836
Bat-CoV-RaTG13_QHR63300.2_MN996532.2                   NTQEVFAQVKQIYKTPPIKDFGGFNFSQILPDPSKPSKRSFIEDLLFNKVTLADAGFIKQ   832
Bat-SARS-like-CoVZC45_AVP78031.1_MG772933.1            NTQEVFAQVKQIYKTPPIKDFGGFNFSQILPDPSKPSKRSFIEDLLFNKVTLADAGFIKQ   809
hCoV-19/ bat/ Yunnan/ PrC31/ 2018| EPI_ISL_1098866     NTQEVFAQVKQIYKTPPIKDFGGFNFSQILPDPSKPSKRSFIEDLLFNKVTLADAGFIKQ   809
hCoV-19/ bat/ Cambodia/ RShSTT182/ 2010| EPI_ISL_852604 NTQEVFAQVKQLYKTPPIKDFGGFNFSQILPDPSKPSKRSFIEDLLFNKVTLADAGFIKQ   814
hCoV-19/ bat/ Cambodia/ RShSTT200/ 2010| EPI_ISL_852605 NTQEVFAQVKQLYKTPPIKDFGGFNFSQILPDPSKPSKRSFIEDLLFNKVTLADAGFIKQ   814
AGT21078.1_SARS_coronavirus_ExoN1                      NTREVFAQVKQMYKTPTLKYFGGFNFSQILPDPLKPTKRSFIEDLLFNKVTLADAGFMKQ   818
AAR86775.1_SARS_coronavirus_ShanghaiQXC2               NTREVFAQVKQMYKTPTLKYFGGFNFSQILPDPLKPTKRSFIEDLLFNKVTLADAGFMKQ   818
                                                       **:********:**** :* ************* **:********************:**
```

```
YP_009724390.1_SARS-COV-2/ Wuhan-Hu-1                       YGDCLGDIAARDLICAQKFNGLTVLPPLLTDEMIAQYTSALLAGTITSGWTFGAGAALQI    896
Bat-CoV-RaTG13_QHR63300.2_MN996532.2                        YGDCLGDIAARDLICAQKFNGLTVLPPLLTDEMIAQYTSALLAGTITSGWTFGAGAALQI    892
Bat-SARS-like-CoVZC45_AVP78031.1_MG772933.1                 YGDCLGGISARDLICAQKFNGLTVLPPLLTDEMIAAYTAALISGTATAGWTFGAGAALQI    869
hCoV-19/ bat/ Yunnan/ PrC31/ 2018| EPI_ISL_1098866          YGDCLGGISARDLICAQKFNGLTVLPPLLTDEMIAAYTAALISGTATAGWTFGAGAALQI    869
hCoV-19/ bat/ Cambodia/ RShSTT182/ 2010| EPI_ISL_852604    YGDCLGDIAARDLICAQKFNGLTVLPPLLTDEMIAQYTSALLAGTITSGWTFGAGAALQI    874
hCoV-19/ bat/ Cambodia/ RShSTT200/ 2010| EPI_ISL_852605    YGDCLGDIAARDLICAQKFNGLTVLPPLLTDEMIAQYTSALLAGTITSGWTFGAGAALQI    874
AGT21078.1_SARS_coronavirus_ExoN1                           YGECLGDINARDLICAQKFNGLTVLPPLLTDDMIAAYTAALVSGTATAGWTFGAGAALQI    878
AAR86775.1_SARS_coronavirus_ShanghaiQXC2                    YGECLGDINARDLICAQKFNGLTVLPPLLTDDMIAAYTAALVSGTATAGWTFGAGAALQI    878
                                                            **:***.* **********************:*** **:**::** *:************

YP_009724390.1_SARS-COV-2/ Wuhan-Hu-1                       PFAMQMAYRFNGIGVTQNVLYENQKLIANQFNSAIGKIQDSLSSTASALGKLQDVVNQNA    956
Bat-CoV-RaTG13_QHR63300.2_MN996532.2                        PFAMQMAYRFNGIGVTQNVLYENQKLIANQFNSAIGKIQDSLSSTASALGKLQDVVNQNA    952
Bat-SARS-like-CoVZC45_AVP78031.1_MG772933.1                 PFAMQMAYRFNGIGVTQNVLYENQKLIANQFNSAIGKIQESLTSTASALGKLQDVVNQNA    929
hCoV-19/ bat/ Yunnan/ PrC31/ 2018| EPI_ISL_1098866          PFAMQMAYRFNGIGVTQNVLYENQKLIANQFNSAIGKIQESLTSTASALGKLQDVVNQNA    929
hCoV-19/ bat/ Cambodia/ RShSTT182/ 2010| EPI_ISL_852604    PFAMQMAYRFNGIGVTQNVLYENQKLIANQFNSAIGKIQDSLSSTASALGKLQDVVNQNA    934
hCoV-19/ bat/ Cambodia/ RShSTT200/ 2010| EPI_ISL_852605    PFAMQMAYRFNGIGVTQNVLYENQKLIANQFNSAIGKIQDSLSSTASALGKLQDVVNQNA    934
AGT21078.1_SARS_coronavirus_ExoN1                           PFAMQMAYRFNGIGVTQNVLYENQKQIANQFNKAISQIQESLTTTSTALGKLQDVVNQNA    938
AAR86775.1_SARS_coronavirus_ShanghaiQXC2                    PFAMQMAYRFNGIGVTQNVLYENQKQIANQFNKAISQIQESLTTTSTALGKLQDVVNQNA    938
                                                            ************************* ******.**.:**:**::*::*************

YP_009724390.1_SARS-COV-2/ Wuhan-Hu-1                       QALNTLVKQLSSNFGAISSVLNDILSRLDKVEAEVQIDRLITGRLQSLQTYVTQQLIRAA    1016
Bat-CoV-RaTG13_QHR63300.2_MN996532.2                        QALNTLVKQLSSNFGAISSVLNDILSRLDKVEAEVQIDRLITGRLQSLQTYVTQQLIRAA    1012
Bat-SARS-like-CoVZC45_AVP78031.1_MG772933.1                 QALNTLVKQLSSNFGAISSVLNDILSRLDKVEAEVQIDRLITGRLQSLQTYVTQQLIRAA    989
hCoV-19/ bat/ Yunnan/ PrC31/ 2018| EPI_ISL_1098866          QALNTLVKQLSSNFGAISSVLNDILSRLDKVEAEVQIDRLITGRLQSLQTYVTQQLIRAA    989
hCoV-19/ bat/ Cambodia/ RShSTT182/ 2010| EPI_ISL_852604    QALNTLVKQLSSNFGAISSVLNDILSRLDKVEAEVQIDRLITGRLQSLQTYVTQQLIRAA    994
hCoV-19/ bat/ Cambodia/ RShSTT200/ 2010| EPI_ISL_852605    QALNTLVKQLSSNFGAISSVLNDILSRLDKVEAEVQIDRLITGRLQSLQTYVTQQLIRAA    994
AGT21078.1_SARS_coronavirus_ExoN1                           QALNTLVKQLSSNFGAISSVLNDILSRLDKVEAEVQIDRLITGRLQSLQTYVTQQLIRAA    998
AAR86775.1_SARS_coronavirus_ShanghaiQXC2                    QALNTLVKQLSSNFGAISSVLNDILSRLDKVEAEVQIDRLITGRLQSLQTYVTQQLIRAA    998
                                                            ************************************************************

YP_009724390.1_SARS-COV-2/ Wuhan-Hu-1                       EIRASANLAATKMSECVLGQSKRVDFCGKGYHLMSFPQSAPHGVVFLHVTYVPAQEKNFT    1076
Bat-CoV-RaTG13_QHR63300.2_MN996532.2                        EIRASANLAATKMSECVLGQSKRVDFCGKGYHLMSFPQSAPHGVVFLHVTYVPAQEKNFT    1072
Bat-SARS-like-CoVZC45_AVP78031.1_MG772933.1                 EIRASANLAATKMSECVLGQSKRVDFCGKGYHLMSFPQSAPHGVVFLHVTYIPSQEKNFT    1049
hCoV-19/ bat/ Yunnan/ PrC31/ 2018| EPI_ISL_1098866          EIRASANLAATKMSECVLGQSKRVDFCGKGYHLMSFPQSAPHGVVFLHVTYIPSQEKNFT    1049
hCoV-19/ bat/ Cambodia/ RShSTT182/ 2010| EPI_ISL_852604    EIRASANLAATKMSECVLGQSKRVDFCGKGYHLMSFPQSAPHGVVFLHVTYVPAQQKNFT    1054
hCoV-19/ bat/ Cambodia/ RShSTT200/ 2010| EPI_ISL_852605    EIRASANLAATKMSECALGQSKRVDFCGKGYHLMSFPQSAPHGVVFLHVTYVPAQQKNFT    1054
AGT21078.1_SARS_coronavirus_ExoN1                           EIRASANLAATKMSECVLGQSKRVDFCGKGYHLMSFPQAAPHGVVFLHVTYVPSQERNFT    1058
AAR86775.1_SARS_coronavirus_ShanghaiQXC2                    EIRASANLAATKMSECVLGQSKRVDFCGKGYHLMSFPQAAPHGVVFLHVTYVPSQERNFT    1058
                                                            ****************.*********************:************:*:*::***

YP_009724390.1_SARS-COV-2/ Wuhan-Hu-1                       TAPAICHDGKAHFPREGVFVSNGTHWFVTQRNFYEPQIITTDNTFVSGNCDVVIGIVNNT    1136
Bat-CoV-RaTG13_QHR63300.2_MN996532.2                        TAPAICHDGKAHFPREGVFVSNGTHWFVTQRNFYEPQIITTDNTFVSGSCDVVIGIVNNT    1132
Bat-SARS-like-CoVZC45_AVP78031.1_MG772933.1                 TAPAICHEGKAHFPREGVFVSNGTHWFVTQRNFYEPKIITTDNTFVSGNCDVVIGIINNT    1109
hCoV-19/ bat/ Yunnan/ PrC31/ 2018| EPI_ISL_1098866          TAPAICHDGKAHFPREGVFVSNGTHWFITQRNFYEPQIITTDNTFVSGNCDVVIGIVNNT    1109
hCoV-19/ bat/ Cambodia/ RShSTT182/ 2010| EPI_ISL_852604    TAPAICHDGKAHFPREGVFVSNGTHWFVTQRNFYEPQIITTDNTFVSGHCDVVIGIVNNT    1114
hCoV-19/ bat/ Cambodia/ RShSTT200/ 2010| EPI_ISL_852605    TAPAICHDGKAHFPREGVFVSNGTHWFVTQRNFYEPQIITTDNTFVSGHCDVVIGIVNNT    1114
AGT21078.1_SARS_coronavirus_ExoN1                           TAPAICHEGKAYFPREGVFVFNGTSWFITQRNFFSPQIITTDNTFVSGNCDVVIGIINNT    1118
AAR86775.1_SARS_coronavirus_ShanghaiQXC2                    TAPAICHEGKAYFPREGVFVFNGTSWFITQRNFFSPQIITTDNTFVSGNCDVVIGIINNT    1118
                                                            *******:***:******** *** **:*****:.*:*********** *******:***

YP_009724390.1_SARS-COV-2/ Wuhan-Hu-1                       VYDPLQPELDSFKEELDKYFKNHTSPDVDLGDISGINASVVNIQKEIDRLNEVAKNLNES    1196
Bat-CoV-RaTG13_QHR63300.2_MN996532.2                        VYDPLQPELDSFKEELDKYFKNHTSPDVDLGDISGINASVVNIQKEIDRLNEVAKNLNES    1192
Bat-SARS-like-CoVZC45_AVP78031.1_MG772933.1                 VYDPLQPELDSFKEELDKYFKNHTSPDIDLGDISGINASVVNIQKEIDRLNEVARNLNES    1169
hCoV-19/ bat/ Yunnan/ PrC31/ 2018| EPI_ISL_1098866          VYDPLQPELDSFKEELDKYFKNHTSPDIDLGDISGINASVVNIQKEIDRLNEVARNLNES    1169
hCoV-19/ bat/ Cambodia/ RShSTT182/ 2010| EPI_ISL_852604    VYDPLQPELDSFKEELDKYFKNHTSPDVDLGDISGINASVVNIQKEIDRLNEVAKNLNES    1174
hCoV-19/ bat/ Cambodia/ RShSTT200/ 2010| EPI_ISL_852605    VYDPLQPELDSFKEELDKYFKNHTSPDVDLGDISGINASVVNIQKEIDRLNEVAKNLNES    1174
AGT21078.1_SARS_coronavirus_ExoN1                           VYDPLQPELDSFKEELDKYFKNHTSPDVDLGDISGINASVVNIQKEIDRLNEVAKNLNES    1178
AAR86775.1_SARS_coronavirus_ShanghaiQXC2                    VYDPLQPELDSFKGELDKYFKNHTSPDVDLGDISGINASVVNIQKEIDRLNEVAKNLNES    1178
                                                            ************* *************:**************************:*****

YP_009724390.1_SARS-COV-2/ Wuhan-Hu-1                       LIDLQELGKYEQYIKWPWYIWLGFIAGLIAIVMVTIMLCCMTSCCSCLKGCCSCGSCCKF    1256
Bat-CoV-RaTG13_QHR63300.2_MN996532.2                        LIDLQELGKYEQYIKWPWYIWLGFIAGLIAIIMVTIMLCCMTSCCSCLKGCCSCGSCCKF    1252
Bat-SARS-like-CoVZC45_AVP78031.1_MG772933.1                 LIDLQELGKYEQYIKWPWYVWLGFIAGLIAIVMVTILLCCMTSCCSCLKGCCSCGSCCKF    1229
hCoV-19/ bat/ Yunnan/ PrC31/ 2018| EPI_ISL_1098866          LIDLQELGKYEQYIKWPWYVWLGFIAGLIAIVMVTILLCCMTSCCSCLKGCCSCGSCCKF    1229
hCoV-19/ bat/ Cambodia/ RShSTT182/ 2010| EPI_ISL_852604    LIDLQELGKYEQYIKWPWYIWLGFIAGLIAIVMVTIMLCCMTSCCSCLKGCCSCGSCCKF    1234
hCoV-19/ bat/ Cambodia/ RShSTT200/ 2010| EPI_ISL_852605    LIDLQELGKYEQYIKWPWYIWLGFIAGLIAIVMVTIMLCCMTSCCSCLKGCCSCGSCCKF    1234
AGT21078.1_SARS_coronavirus_ExoN1                           LIDLQELGKYEQYIKWPWYVWLGFIAGLIAIVMVTILLCCMTSCCSCLKGACSCGSCCKF    1238
AAR86775.1_SARS_coronavirus_ShanghaiQXC2                    LIDLQELGKYEQYIKWPWYVWLGFIAGLIAIVMVTILLCCMTSCCSCLKGACSCGSCCKF    1238
                                                            *******************:***********:****:*****:*****************

AGT21078.1_SARS_coronavirus_ExoN1                           DEDDSEPVLKGVKLHYT     1255
AAR86775.1_SARS_coronavirus_ShanghaiQXC2                    DEDDSEPVLKGVKLHYT     1255
Bat-SARS-like-CoVZXC21_AVP78042.1                           DEDDSEPVLKGVKLHYT     1245
Bat-SARS-like-CoVZC45_AVP78031.1_MG772933.1                 DEDDSEPVLKGVKLHYT     1246
hCoV-19/ bat/ Yunnan/ PrC31/ 2018| EPI_ISL_1098866          DEDDSEPVLKGVKLHYT     1246
hCoV-19/ bat/ Cambodia/ RShSTT182/ 2010| EPI_ISL_852604    DEDDSEPVLKGVKLHYT     1251
hCoV-19/ bat/ Cambodia/ RShSTT200/ 2010| EPI_ISL_852605    DEDDSEPVLKGVKLHYT     1251
YP_009724390.1_SARS-COV-2/ Wuhan-Hu-1                       DEDDSEPVLKGVKLHYT     1273
Bat-CoV-RaTG13_QHR63300.2_MN996532.2                        DEDDSEPVLKGVKLHYT     1269
                                                            *****************
```
