# Supplementary material for: Conserved molecular signatures in the spike protein provide evidence indicating the origin of SARS-CoV-2 and a Pangolin-CoV (MP789) by recombination(s) between specific lineages of Sarbecoviruses
Source: PeerJ. 2021 Nov 12;9:e12434. doi: 10.7717/peerj.12434 (PMC8592051; doi:10.7717/peerj.12434)
Supplement: Supplemental Information 6 — This Spp1 restriction site is also present (highlighted) in the sequences of other Sarbecoviruses and it is indicated to be the breakpoint in the two recombination events described in this work. [file peerj-09-12434-s006.pdf]

Ssp1 site

caaccaactgaatctatagttaggtttccaaatattacaaacttatgcccttttggtgaag

317 Q P T E S I V R F P N I T N L C P F G E 336

	988		1048
Wuhan-Hu-1_NC_045512.2	CAACCAACAGAATCTATTGTTAGATTTCCT	AATATT	ACAAACTTGTGCCCTTTTGGTGAAG
Bat-CoV-RaTG13_MN996532.2	CAACCAACAGATTCTATTGTTAGATTCCCA	AATATT	ACAAACTTATGTCCTTTTGGTGAAG
Pangolin-CoV_MP789_MT121216.1	CAACCAACTGAATCTATAGTTAGGTTTCCA	AATATT	ACAAACTTATGCCCTTTTGGTGAAG
Bat-SARS-like-CoVZXC21_MG772934.1	CAACCCACTCAGTCTATAGTTAGATTTCCT	AATATT	ACCAATGTGTGTCCATTTCACAAGG
Bat_SARS-lik_CoV_ isolate bat-SL-CoVZC45_MG772933.1	CAACCCACTCAGTCTGTAGTTAGATTTCCT	AATATT	ACCAATGTGTGTCCATTTCACAAGG
hCoV-19batYunnanPrC312018_EPI_ISL_1098866	CAACCCACTCAGTCAGTAGTTAGATTTCCT	AATATT	ACCAATGTGTGTCCTTTTCACAAGG
hCoV-19batCambodiaRShSTT182_EPI_ISL_852604	TCTCCTACTACACAAGTTGTTAGGTTTCCT	AATATT	ACAAATTTATGCCCTTTTGGTGAAG
hCoV-19batCambodiaRShSTT200_EPI_ISL_852605	TCTCCTACTACACAAGTTGTTAGGTTTCCT	AATATT	ACAAATTTATGCCCTTTTGGTGAAG
hCoV-19batYunnanRmYN022019_EPI_ISL_412977	TTGCCTTCTACAGAAGTGGTGAGATTTCCT	AATATT	ACAAACTTTTGCCCATTTGACAAGG
Bat_SC2r-CoV RacCS203_Thailand_2020_MW251308.1	TTGCCCTCTACAGAAGTGGTGAGATTTCCT	AATATT	ACAAACTTTTGCCCATTTGACAAGG
Bat_SC2r-CoVRc-o319_Japan_2013	CAACCACAAGATACGGTTGTGAGGTTCCCT	AATATT	ACAAACCTTTGTCCTTTTTCAGAGG
SARS CoV-ExoN1_FJ882930.1	GTTCCCTCAGGAGATGTTGTGAGATTCCCT	AATATT	ACAAACTTGTGTCCTTTTGGAGAGG

Figure S6. Nucleotide and amino acid sequence of the spike protein from pangolin-CoV_MP789 surrounding the site where sequence changes from CoVZC-like to SARS-CoV-2 like sequence. This Spp1 restriction site is also presented in the sequences of other Sarbecoviruses.
