# Supplementary material for: Conserved molecular signatures in the spike protein provide evidence indicating the origin of SARS-CoV-2 and a Pangolin-CoV (MP789) by recombination(s) between specific lineages of Sarbecoviruses
Source: PeerJ. 2021 Nov 12;9:e12434. doi: 10.7717/peerj.12434 (PMC8592051; doi:10.7717/peerj.12434)
Supplement: Supplemental Information 7 — The location of identified 1 aa deletion is highlighted. Detailed species distribution information for the 1 aa deletion in Fig. S2A). (B) Homology model of RBD of SARS-related coronavirus BtKY72 spike protein (Acc no: APO40579) based on available experimental structure of SARS coronavirus BJ012 spike protein (PDB: 5x58). The receptor binding domain (RBD) domain is highlighted as green and extended C-terminal region as pale green. The locations of identified signature indels are highlighted red and labelled. Detailed species distribution information for the 1 aa insert and 2 aa insert in RBD in Fig. S2B) and Fig. S2C. [file peerj-09-12434-s007.pdf]

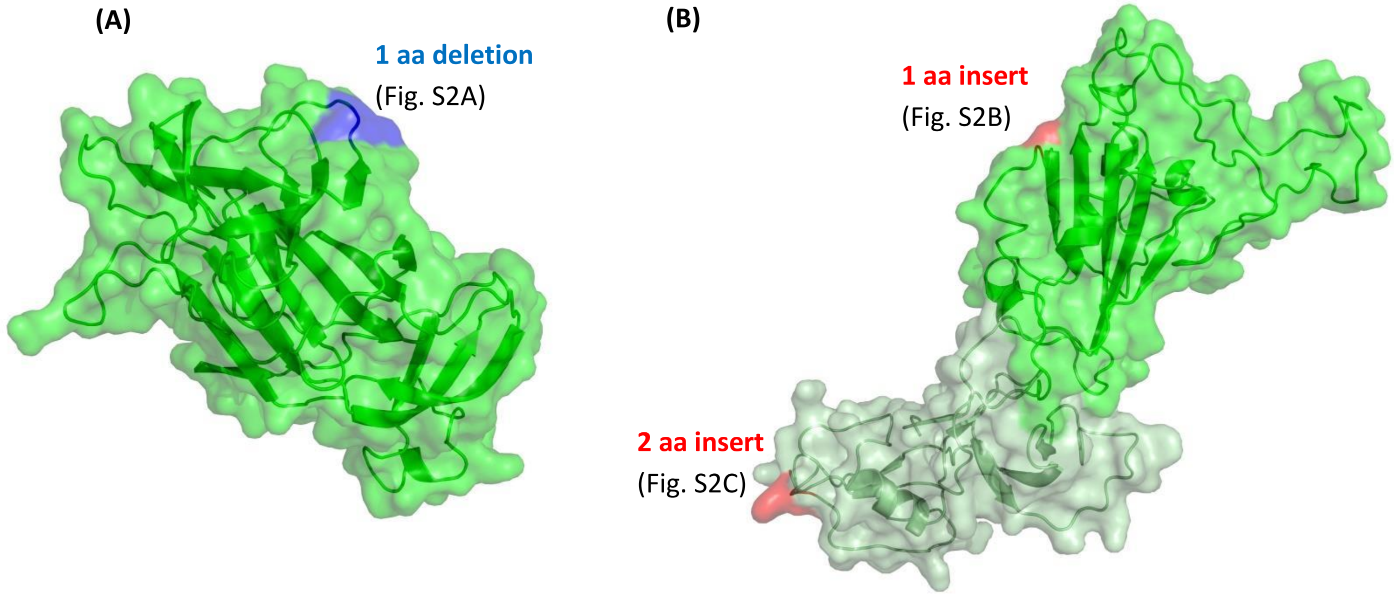

Figure S7. (A). Homology model of N-terminal domain (NTD) of spike protein from SARS-related coronavirus BtKY72 (Acc no: APO40579) based on available experimental structure of SARS coronavirus BJ012 spike protein (PDB: 5x58). The location of identified 1 aa deletion is highlighted. Detailed species distribution information for the 1 aa deletion in Figure S2A. (B). Homology model of RBD of SARS-related coronavirus BtKY72 spike protein (Acc no: APO40579) based on available experimental structure of SARS coronavirus BJ012 spike protein (PDB: 5x58). The receptor binding domain (RBD) domain is highlighted as green and extended C-terminal region as pale green. The locations of identified signature indels are highlighted red and labelled. Detailed species distribution information for the 1 aa insert and 2 aa insert in RBD in Figure S2B and S2C.
